# Effects of Phenylephrine Administration on the Circulatory Dynamics of Patients with Hypotension Due to Bleeding During Surgery, Specifically Left Ventricular End-Diastolic Volume, Effective Arterial Elastance, and Left Ventricular End-Systolic Elastance

**DOI:** 10.3390/jcm15020905

**Published:** 2026-01-22

**Authors:** Takahiro Shiraishi, Mayuki Sato, Rina Takagi, Kenji Shigemi, Yuka Matsuki

**Affiliations:** 1 Department of Anesthesiology and Reanimatology, University of Fukui Hospital, Fukui 910-1193, Japan; ymatsuki@u-fukui.ac.jp; 2 School of Medical Sciences, University of Fukui, Fukui 910-1193, Japan; st23049@g.u-fukui.ac.jp (M.S.); st23055@g.u-fukui.ac.jp (R.T.); 3 Maizuru Municipal Hospital, Kyoto 625-8555, Japan; kshigemi@hospital.maizuru.kyoto.jp

**Keywords:** left ventricular end-diastolic volume, Ees/Ea, preload, stroke volume

## Abstract

**Background/Objectives**: Under general anesthesia, maintaining patients’ blood pressure (BP) is important to prevent organ ischemia. When bleeding occurs, it is sometimes difficult to increase BP with boluses of fluids or transfusions, and vasoconstrictors must be administered. This study investigated circulatory dynamic changes in patients who bled during surgery and were administered phenylephrine, particularly left ventricular end-diastolic volume (EDV), effective arterial elastance (Ea), and left ventricular end-systolic elastance (Ees), calculating each value from the left ventricular–arterial coupling (Ees/Ea). **Methods**: We calculated Ees/Ea using electrocardiograms, arterial pressure waveforms, and phonocardiograms using an esophageal stethoscope. We investigated the changes in patients’ EDV, Ea, and Ees during two periods: phenylephrine administration and after BP elevation. **Results**: The seven participants comprised three men and four women. Between the two periods, linear mixed-model analysis revealed that mean arterial pressure (MAP), Ea, and Ees significantly increased over time (MAP; β = 8.7, *p* < 0.01, Ea; β = 0.22, *p* < 0.05, Ees; β = 0.73, *p* < 0.05), while no significant changes were observed in other parameters such as heart rate and EDV. Conventional parameters demonstrated that stroke volume variation significantly decreased (β = −2.0, *p* = 0.01), systemic vascular resistance index significantly increased (β = 200, *p* < 0.01), while no significant change was observed in cardiac index (β = −0.03, *p* = 0.7). In patients administered phenylephrine due to BP decrease from bleeding, significant changes in afterload and cardiac contractility occurred without changes in preload. **Conclusions**: Our noninvasive method for calculating EDV, Ea, and Ees can be valuable for monitoring hemodynamics under anesthesia.

## 1. Introduction

This study is conducted with the aim of developing a less invasive method for evaluating hemodynamics in patients under general anesthesia. The underlying concept involves hemodynamic assessment using left ventricular–aortic coupling, and historically, the focus has been on how this coupling changes due to bleeding or sympathetic stimulation. However, individual variations in parameters derived from left ventricular–aortic coupling—specifically, end-diastolic volume, effective aortic compliance, and end-systolic compliance—have not been analyzed. By analyzing each parameter separately, we can independently assess left ventricular preload, afterload, and contractility. We conducted this study because we believe this approach provides clearer and more meaningful insights than previous evaluations of left ventricular–aortic coupling.

When general anesthesia is administered to surgical patients, blood pressure often decreases because the anesthetic drugs enhance parasympathetic nervous system dominance. Therefore, maintaining stable blood pressure is crucial to prevent organ ischemia. It is reported that “organ injury might occur when mean arterial pressure decreases <80 mmHg for ≧10 min, and that this risk increases with blood pressures becoming progressively lower.” [1]. The optimal blood pressure level varies among patients, and some consider it desirable to maintain values close to those measured in daily life without anesthesia. Futier and colleagues reported that in patients predominantly undergoing abdominal surgery who were at increased postoperative risk, management targeting individualized systolic blood pressure, compared with standard management, reduced the risk of postoperative organ dysfunction [2]. To achieve stable blood pressure, left ventricular preload, afterload, and contractility must be appropriately controlled.

Classically, left ventricular preload, afterload, and contractility have been estimated using left ventricular end-diastolic volume (EDV) measurement with transesophageal echocardiography (TEE) and methods to calculate the systemic vascular resistance index (SVRI) and cardiac index (CI) derived from stroke volume measurements taken using a Swan–Ganz catheter. These have been the most reliable methods [3]; however, they are highly invasive, to the extent that they are no longer easily used in noncardiac surgery. Recently, the Flo Trac^TM^ system (Edwards Lifesciences, Irvine, CA, USA; currently BD Advanced Patient Monitoring following the acquisition of Edwards’ Critical Care business), which only requires an arterial line, has been widely adopted to continuously estimate parameters such as stroke volume variation (SVV), SVRI, and CI. Anesthesia is controlled using these parameters as a guide. Although these parameters are highly useful, they have certain limitations; specifically, SVV is a relative rather than an absolute value; thus, it is not possible to estimate the precise amount of preload from the SVV value alone. The SVRI does not indicate pure afterload because it includes peripheral vascular resistance. CI is considered reliable; however, it is derived from the stroke volume (SV) and is not only a parameter for heart contractility itself but also the preload. Vincent and his colleagues stated that “Cardiac index is a global flow parameter determined by the interplay of preload, cardiac contractility, afterload, and heart rate, and thus serves as an indicator of overall cardiac function rather than a specific measure of myocardial contractility or volume status alone.” [4]. These parameters should be evaluated in combination rather than individually, which makes them somewhat difficult to grasp intuitively.

**Figure 1:**
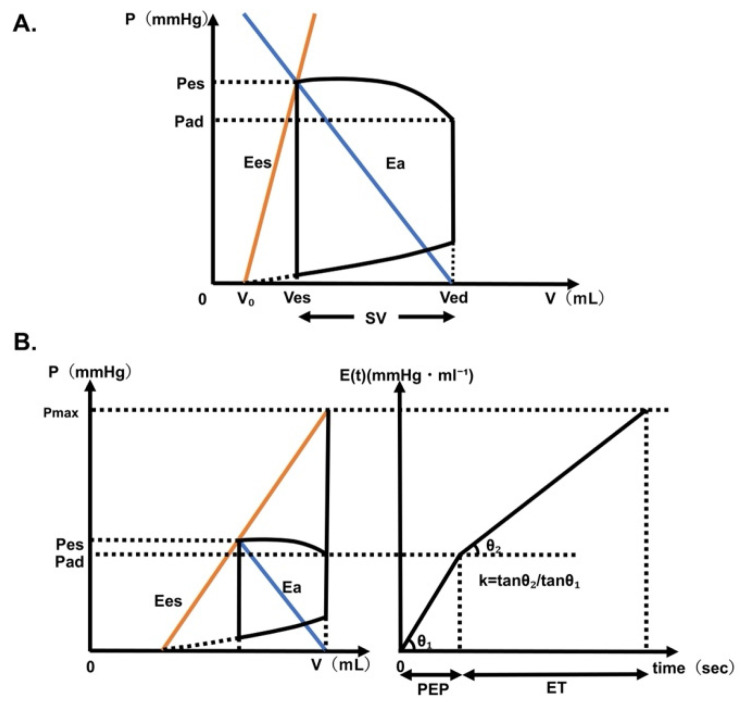
Left ventricular pressure–volume trajectory and left ventricular time-varying elastance. (**A**) The left ventricular pressure–volume trajectory is illustrated. EDV, Ea, and Ees can be calculated using left ventricular–arterial coupling (Ees/Ea). Ees/Ea represents the balance between the left ventricle and the aorta that receives its output. These three parameters can be calculated using the following equations: EDV = SV·{1 + (Ees/Ea)^−1^} + V_0_, Ea = Pes/SV, Ees = Pes/(ESV − V_0_), where Pes: end-systolic pressure; Pad: diastolic pressure; Pmax: putative isovolumic pressure; SV: stroke volume; and V_0_ is the volume when the left ventricular pressure is zero. (**B**) Left: trajectory graph for the pressure–volume curve during a single heartbeat. Right: Graph for two linear approximation lines of time-varying elastance {E(t)}; one is the approximation of the PEP, and the other is of the systolic phase. The trajectory on the pressure–volume curve during left ventricular systole can be interpreted as an increase in the elastance [E(t) = P(t)/(V(t) − V0)]. The left ventricular time-dependent elastance E(t) represents the temporal progression of left ventricular elastance during a single heartbeat. E(t) during the systolic phase is known to show a distinctive trajectory. The slope ratio of the two approximation lines of PEP and the systolic period is defined as constant(k). The ratio of the slopes of those two lines (tanθ1/tanθ2) can be shown with a constant(k). The constant(k) is needed to obtain Ees/Ea using the method by Hayashi et al. [5]. Ea, effective arterial elastance; EDV, end-diastolic volume; Ees, end-systolic elastance; ESV, end-systolic volume; ET, ejection time; PEP: pre-ejection period.

To overcome this limitation, we developed a less invasive method for estimating left ventricular preload, afterload, and heart contractility, which would otherwise require TEE or a Swan–Ganz catheter. This method combines electrocardiogram, arterial pressure waveform, and phonocardiogram data. Phonocardiogram results are obtained using an orally inserted esophageal stethoscope. From these signals, the pre-ejection period (PEP) and ejection time (ET) can be derived, which, together with Hayashi’s method, enable calculation of left ventricular–arterial coupling (Ees/Ea) [5]. Ees/Ea represents the balance between the left ventricle and the aorta that receives its output. This is expressed as the ratio of Ees to Ea in the left ventricular pressure–volume relationship (Figure 1A). Ees represents left ventricular contraction, whereas Ea represents left ventricular afterload. Using this approach, only an arterial line and esophageal stethoscope are required, making it far less invasive than TEE. Furthermore, left ventricular EDV can be calculated by combining Ees/Ea with the SV obtained from the FloTrac™ system.

In this study, we investigated how the EDV, Ea, and Ees calculated using this method changed in response to phenylephrine administration during bleeding-induced hypotension under general anesthesia. When bleeding occurs, anesthesiologists typically attempt to restore blood pressure with fluid resuscitation or transfusion. However, rapid infusion alone is sometimes insufficient. Russell and his colleague reported that “Among critically ill adults undergoing tracheal intubation, administration of an intravenous fluid bolus compared with no fluid bolus did not significantly decrease the incidence of cardiovascular collapse.” [6]. In contrast, vasopressors are effective in mobilizing venous blood toward the heart, thereby increasing the preload.

Phenylephrine is a pure α-adrenergic agonist with potent vasoconstrictive effects on peripheral veins. When administered during bleeding, it is expected to increase the afterload and possibly the preload, although the latter effect may depend on the extent of blood loss. In this study, we evaluated the changes in EDV, Ea, and Ees estimated using our noninvasive method in this clinical context and observed whether they followed the actual hemodynamic changes in a patient’s body. In other words, we sought to capture circulatory fluctuations caused by vasopressor administration during bleeding and assess whether preload, afterload, and contractility could be monitored more noninvasively, facilitating broader clinical application.

## 2. Materials and Methods

### 2.1. Ethics Approval and Participants

This prospective observational study was approved by the Ethics Review Committee of Fukui University (approval number 20220063; date of approval 2 August 2022) and conducted in accordance with the Declaration of Helsinki. Written informed consent was obtained from all participants. All participants were adults with normal cardiac function. Patients with cardiovascular diseases, including vasculitis, myocardial infarction, and chronic heart failure, were excluded. This study was conducted at the University of Fukui Hospital between February 2024 and August 2024.

### 2.2. Equipment and Procedure

**Figure 2:**
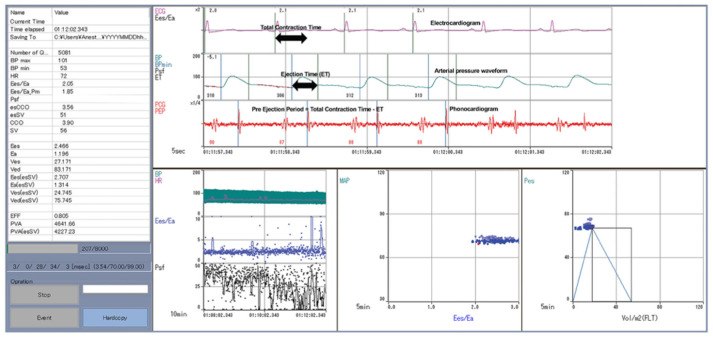
Measuring device screen.

Electrocardiograms, radial arterial pressure waveforms, and phonocardiograms were recorded. All monitors were connected to a CSM-1000 series, Life Scope G7 (NIHON KOHDEN CORPORATION, Shinjuku, Tokyo, Japan), and all data were collected and stored in a PRM-7500 system (Prime Gaia^TM^, NIHON KOHDEN COPORATION, Tokyo, Japan). After anesthesia induction, a radial artery catheter was placed and connected to a Flo Trac sensor (Edwards Lifesciences, Irvine, CA, USA), and vital data were collected on the EV1000 Clinical Platform^TM^ (Edwards Lifesciences, Irvine, CA, USA) to measure and display SV. All the data obtained from the monitors were collected on a laptop containing the measuring software. The measurement software and laptop were provided by NIHON COHDEN CORPORATION. The screen of the measuring device is shown in Figure 2. ET was defined as the pure ejection phase of the left ventricular systole, measured as the interval between the onset of contraction and the dicrotic notch on the radial artery pressure waveform. An esophageal stethoscope was orally inserted to monitor the heart sounds and identify the second heart sound (S2). The heart sounds were amplified using a condenser microphone connected to an esophageal stethoscope and imported into a computer. The heart sounds were visualized using an analysis software, and S2 was identified as the closure of the aortic and pulmonary valves. The PEP was measured using S2. The total duration of contraction was calculated as the interval between the onset of the QRS complex on the electrocardiogram and the occurrence of S2 on the phonocardiogram. The PEP was obtained by subtracting ET from the total contraction duration. Left ventricular–aortic coupling (Ees/Ea) was calculated using end-systolic pressure (Pes), diastolic pressure (Pad), PEP, and ET. This method of calculating Ees/Ea follows Hayashi’s method [4]. The Pes was calculated using the correction equation proposed by Kappus et al. [7].

### 2.3. Deriving Ees/Ea with Hayashi’s Method

Hayashi’s method comprises theoretical equations and experimental values [4]. Left ventricular elastance (E(t)) was linearly approximated during the isovolumic contraction and ejection phases, and the resulting slope ratio (rate of decrease in left ventricular elastance) was defined as k (Figure 1). In this context, neither the diastolic phase nor the heart rate was considered. The hypothetical left ventricular end-systolic pressure (peak isovolumic pressure; [Pmax]), under the condition of the aorta being clamped and without ventricular ejection occurring, could be expressed using PEP, ET, and Pad, as follows:

Pmax = Pad + Pad·(ET/PEP)·k = Pad·{1 + (ET/PEP)·k}(1)

The increase in left ventricular pressure from Pes to Pmax, and the increase in aortic pressure from Pes, were both attributable to the same left ventricular SV. Accordingly, the Ees/Ea ratio was expressed as follows:

Ees/Ea = (Pmax − Pes)/Pes(2)

From Equations (1) and (2), the following theoretical equation is derived:

Ees/Ea = Pad/Pes·(1 + k·ET/PEP) − 1(3)

Thus, by measuring arterial pressure and systolic time intervals, the Ees/Ea ratio could be obtained from the theoretical equation, eliminating the need for the direct measurement of left ventricular volume. In a previous study [4], the value of k was determined experimentally, and the following regression equation was obtained:

k = 0.53·(Ees/Ea)^0.51^ (4)

The experimental equation was substituted into the theoretical formula to eliminate k, thereby obtaining an expression for Ees/Ea. By substituting the measured values of the ET/PEP and Pad/Pes into this equation and solving it using Newton’s method, the estimated value of Ees/Ea was obtained.

### 2.4. Method for Deriving EDV, Ea, and Ees from Ees/Ea

From the pressure–volume (PV) loop diagram, Ea can be calculated geometrically. Combining this with the calculated value of Ees/Ea obtained from the PEP and other measurements allows Ea and Ees to be derived from the following equations:

Ea = Pes/SV(5)

Ees = Ees/Ea·Ea(6)

Furthermore, from the PV loop diagram, Ees can be expressed as follows:

Ees = Pes/(EDV − SV − V_0_) (7)

By substituting Equations (5) and (7) into Ees/Ea,

Ees/Ea = SV/(EDV − SV − V_0_) (8)

EDV = SV·(1 + Ea/Ees) + V_0_ (9)

V_0_ is considered negligible and approximated as 0 for patients with normal cardiac function because it is very small compared to the EDV and ESV. Under this assumption, the EDV can be expressed as follows:

EDV = SV·(1 + Ea/Ees)(10)

Thus, the EDV is calculated by substituting the SV obtained from the FloTrac sensor into the above equations. From these relationships, the EDV is derived as a function of the SV and Ees/Ea (Ea/Ees is the reciprocal of Ees/Ea).

### 2.5. Data Sampling Method

The time points at which blood pressure declined during general anesthesia were extracted, and the presence of bleeding at those points was verified using the anesthesia records. If bleeding occurred within 30 min before or after the extracted time point, the decline in blood pressure was attributed to bleeding.

Hypotension was defined based on the mean arterial pressure (MAP). The patients who were administered a single injection via the epidural catheter within 1 h of the time point selected for identifying hypotension were excluded, because the effect of epidural anesthesia on blood pressure could not be ruled out. To isolate the effects of phenylephrine, patients administered other pressor agents at the time of data collection were excluded. Among the patients in whom continuous pressor agents were used, those with a significant increase or decrease in the infusion dose within 30 min before or after data collection were excluded.

For comparisons, data were collected at two time points: during the hypotensive phase (defined as the average value during the 5 min immediately preceding phenylephrine administration) and during the late vasopressor phase (defined as the average value during the 5 min following the 5 min mark after phenylephrine administration), and the timing for data collection after administration was determined by considering the onset time and duration of phenylephrine’s effect. The onset time for phenylephrine is 1 to 3 min, and its duration of effect is considered to be 10 to 20 min [8]. Therefore, 5 min after administration is considered a time when the effect is fully manifested. Furthermore, 5 to 10 min after administration was considered the time when the effect of phenylephrine was maximal. At these two time points, eight parameters were analyzed: heart rate (HR), MAP, SVV, SVRI, CI, EDV, Ea, and Ees.

The changes before and after phenylephrine administration were assessed using a linear mixed-effects model to account for individual variability. EDV, Ea, and Ees were normalized using body surface area, which was calculated using the Du Bois formula [9].

### 2.6. Statistical Analyses

Statistical analysis was performed using R (ver. 4.5.2) and the Ime4, ImerTest, and MuMIn packages. Each indicator analyzed (HR, MAP, SVV, SVRI, CI, EDV, Ea, and Ees) was selected as a dependent variable. Linear mixed-effects models were constructed individually for each parameter. The model structure was common across all, with age, gender, blood loss, presence of hypertension, and measurement time set as fixed effects. To account for inter-subject variability, patient ID was used as a random effect (random intercept). Restricted maximum likelihood (REML) was used for parameter estimation, and degrees of freedom were estimated using Satterthwaite’s approximation for *p*-value calculation. Based on Nakagawa’s method, we calculated Marginal R^2^, indicating the explanatory power of fixed effects alone, and Conditional R^2^, indicating the overall explanatory power of the model including random effects.

For each patient who developed hypotension due to bleeding, we identified the point at which the blood pressure increased following phenylephrine administration. We compared the mean values of HR, MAP, SVV, SVRI, CI, EDV, Ea, and Ees between the hypotensive phase (preceding phenylephrine administration) and the post-pressor phase (following administration). The results from all the patients were pooled for statistical analyses. Based on SVRI, a conventional indicator of afterload, the required sample size was calculated using the expected difference in the SVRI. The change in the SVRI before and after phenylephrine administration was extracted from the pilot data, yielding an effect size of 0.76. The required sample size was estimated using G*Power (Version 3.1.9.6). Assuming a two-sided *t*-test with an α error of 0.05, a power of 0.8, and a dropout rate of 20%, the calculated sample size was 20. Although only seven patients were enrolled in this study, multiple datasets were extracted for each patient to compensate for the limited sample size. For individual patients, the number of extracted datasets ranged from two to five. Accordingly, statistical analyses were performed using these datasets as the unit of analysis rather than using individual patients. And therefore, we decided to use linear mixed-model analysis, taking into an account that there was an effect caused by multiple attempts from the same patient.

## 3. Results

### 3.1. Patient Characteristics

The patient characteristics are listed in Table 1. The seven patients comprised three men and four women (mean age 61 ± 9 years, mean height 160 ± 6 cm, and mean weight 60 ± 9 kg).

The number of datasets per patient ranged from 2 to 5, resulting in 20 datasets. The mean values were as follows: PEP, 87 ± 12 s; ET, 298 ± 16 s; EDV, 49 ± 6.3 mL/m^2^; Ea, 2.2 ± 0.4 mmHg·m^2^/mL; Ees, 5.5 ± 1.4 mmHg·m^2^/mL; and SV, 54 ± 7 mL.

Total intravenous anesthesia was used for five patients, all of whom also received epidural anesthesia. Two patients received inhalation anesthesia, one of whom also received epidural anesthesia and the other received regional anesthesia for analgesia. All the patients had a class II American Society of Anesthesiologists physical status.

### 3.2. Representative Case

**Figure 3:**
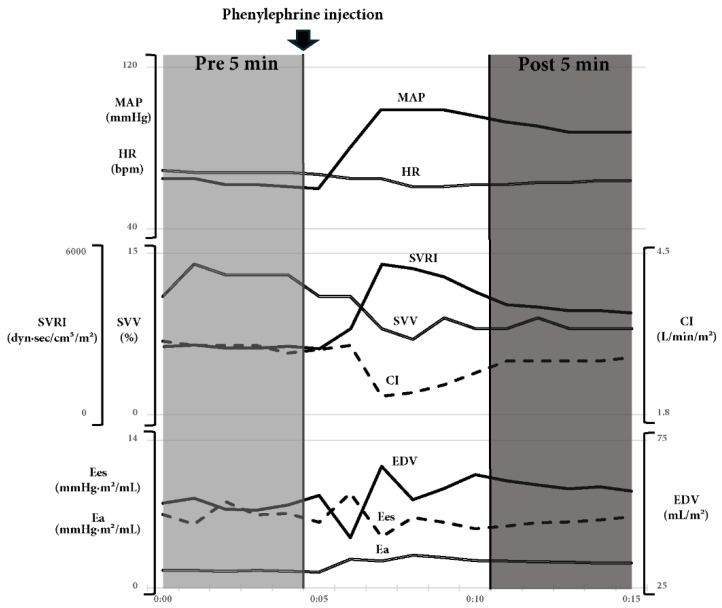
Representative time course of a patient who was administered phenylephrine to restore blood pressure following bleeding during surgery. The figure shows the timing of the vasopressor administration and the two data collection points. The timing of phenylephrine administration is indicated by an arrow, and the two data collection points are indicated by colored boxes. In this case, phenylephrine was administered at the point where the MAP decreased. Subsequently, an increase in the SVRI and Ea, both indicators of afterload, was observed, whereas no changes were observed in the indicators of preload or contractility. CI, cardiac index; Ea, effective arterial elastance; EDV, end-diastolic volume; Ees, end-systolic elastance; HR, heart rate; MAP, mean arterial pressure; SVRI, systemic vascular resistance index; SVV, stroke volume variation.

Figure 3 shows a representative case of phenylephrine administration. In this case, 50 µg of phenylephrine were administered to a 47-year-old woman with a BMI of 23 kg/m^2^ who underwent a total hysterectomy. At the time of data sampling, 560 mL of blood had been lost. Blood pressure increased, accompanied by elevations in EDV and Ea, whereas Ees showed little change. Regarding the conventional indicators, SVV decreased, SVRI increased, and CI remained unchanged. As in this patient, we predicted that phenylephrine would result in either an increase or no change in preload and afterload, whereas heart contractility would remain unchanged in patients with hypovolemia due to bleeding.

### 3.3. Linear Mixed-Effect Model Analysis

The results of the linear mixed-effect model analysis for each indicator are shown in Table 2. Examination of the fixed effect for measurement time revealed significant changes in MAP, SVRI, SVV, Ea, and Ees. Ea showed a significant positive association with measurement time (β = 0.22, SE = 0.11, *p* < 0.05), and Ees showed a significant positive association (β = 0.73, SE = 0.36, *p* < 0.05). In contrast, no significant effect of measurement time was observed for EDV. No significant effects were observed for HR or CI either (all *p* > 0.05). Regarding model fit, Marginal R^2^ ranged from 0.08 to 0.35, and Conditional R^2^ ranged from 0.31 to 0.88. Detailed estimates and fit indices for each indicator are shown in the table.

## 4. Discussion

In this study, we investigated hemodynamic changes induced by phenylephrine administration in patients who developed hypotension due to bleeding under general anesthesia during surgery. Three parameters were the focus: EDV, Ea, and Ees. Phenylephrine significantly increased Ea, an indicator of left ventricular afterload, and Ees, an indicator of cardiac contractility, whereas EDV, which represents preload, remained unchanged. An increase in Ea (equivalent to an increase in afterload) was followed by phenylephrine administration. However, phenylephrine does not usually increase the Ees (equivalent to an increase in heart contractility). It is well known that “Phenylephrine primarily acts as an alpha-1 adrenergic receptor agonist and exhibits minimal to no beta-adrenergic activity.” [10]. Phenylephrine is a sympathomimetic alpha-receptor agonist possessing only peripheral vasoconstrictive effects, and the discrepancy between this and the outcomes it should induce requires consideration. We first examined the hemodynamic changes associated with bleeding in patients during general anesthesia and then confirmed the mechanism of action of phenylephrine. Subsequently, we systematically examined the hemodynamic changes that we originally anticipated and the reasons these predictions differed from the results obtained in this study.

### 4.1. Predicted Hemodynamic Changes

In this study, phenylephrine was administered to treat bleeding-induced hypotension under general anesthesia. In bleeding situations, the preload is typically decreased, and a reduction in the circulating volume may lead to a compensatory increase in contractility. In hemorrhagic shock situations, the body compensates for volume loss by increasing the heart rate and contractility. As diastolic ventricular filling continues to decline and cardiac output decreases, systolic blood pressure drops [11]. The afterload is normally maintained or even elevated, and general anesthesia can lower the afterload due to vasodilation.

Phenylephrine is a pure α-adrenergic agonist with peripheral vasoconstrictive effects. Therefore, its administration is expected to increase afterload, and through venoconstriction, potentially augment venous return and thereby increase preload without affecting cardiac contractility. Depending on the preload dependency, phenylephrine boluses induce a decrease in cardiac output when the heart is preload-independent. Additionally, when the heart is preload-dependent, phenylephrine boluses induce an increase in cardiac output. These changes in cardiac output are closely related to changes in venous flow induced by phenylephrine administration [12].

We anticipated that phenylephrine administration under this condition would result in an increase in afterload and possibly an increase in preload, with little or no change in contractility. Contrary to this expectation, our results showed that although afterload increased, preload did not change, and contractility unexpectedly increased. In this study, only the increase in afterload was consistent with the expected pharmacological effect of phenylephrine, whereas the changes in preload and contractility differed from the initial assumptions. However, these discrepancies may be explained by the combined effects of phenylephrine and bleeding-induced hemodynamic alterations.

### 4.2. Interpretation of the Findings: Preload

Regarding preload, bleeding likely reduced venous return, and the maintenance of preload may have depended on the shift from an unstressed volume to a stressed volume within the venous system. It is traditionally explained as a preload dependency in a Frank–Starling curve, and in this state, fluid therapy improves venous return significantly. Phenylephrine also improves this state by converting unstressed volume into stressed volume, but it depends on how much unstressed volume is reserved in patients’ bodies. Consequently, when phenylephrine was administered, its venoconstrictive effect could not further increase the preload, because the available reserve was already limited. The extent of bleeding and the amount of fluid or blood transfusion administered at the time of measurement may also have influenced the results. If the balance is too hypovolemic, which is caused by insufficient infusion or transfusion, the reserved volume could be too low to afford the venous return. A study that investigated the effect of phenylephrine on preload dependency induced by the head-up tilt position found that phenylephrine administration increased preload, as measured by SVV [13]. Possibly, in cases with minimal bleeding, EDV would have increased following phenylephrine administration. And with this context, we can determine if we are able to fight against hypovolemia just to administrate phenylephrine, like through a restrictive fluid therapy, or if we should add more volume, infusion, or transfusion products. If EDV is continuously obtained and presented on the vital monitor, it could help our strategy to treat patients with either vasoconstrictor or infusion/transfusion therapy, or both.

Our method can evaluate EDV with high accuracy and is expected to be utilized as an indicator for hemodynamic monitoring in the future. On the other hand, based on these results, it is necessary to consider whether EDV measurement is truly suitable for clinical practice. In this regard, it is necessary to refer to and consider the paper by Giraud et al. [14]. They demonstrated the effects of pressor agents on PPV and SVV as markers for inferior vena cava flow by artificially making pigs hypovolemic, and concluded that “Intravenous administrations of norepinephrine and epinephrine increase IVCF, whatever the volemic conditions are.” [14]. This is a controversial result compared to our results. It could lead to the conclusion that our method, EDV measurement and its assessment, is not appropriate for the understanding of preload. However, it was norepinephrine and epinephrine, not phenylephrine, that was used for the study, and those drugs act as beta-adrenergic agonists in addition to having their alpha-adrenergic effects. That is one of the differential points from our study, and furthermore, the authors induced a hypovolemic status in pigs artificially. That does not always reflect the actual clinical situation. Although there is a possibility that we could not accurately calculate EDV, and phenylephrine may have had an autotransfusion-like effect due to recruitment from the venous system even leading to hypovolemia, the previous study did not isolate the inotropic effect, which could have been problematic.

### 4.3. Interpretation of the Findings: Contractility

As for the increase in contractility, several hypotheses are plausible. Firstly, it could be a physiological response to hypotension associated with bleeding rather than a direct inotropic effect of phenylephrine. When bleeding occurs, venous return decreases and baroreceptor reflex causes physiological changes in a patient’s body, activating the sympathetic nerve. Heart contractility increases and HR also increases in order to compensate for the decrease in SV and to preserve cardiac output and MAP. Afterload should be increased at first, but when hypotension occurs, compensatory mechanisms likely reach their limits, and afterload is thought to be decreasing. According to the results presented in Table 2, it could be understandable that the increase in Ees could be explained as the compensatory change due to hemorrhagic shock.

Secondly, it is possible that the significant increase in afterload led to an increase in contractility. Sequeira et al. explained this with the Anrep effect in the context of septic shock [15]. Although they talked about norepinephrine, the fact that the massive increase in afterload made the contractility increase afterwords could be appropriate for phenylephrine as well. In fact, despite the observed increase in myocardial contractility, conventional indices such as CI remained unchanged. CI is explained by the multiplication of HR and SV, so the fact that CI remained unchanged could be equivalent to the fact that SV remained unchanged. The result that EDV did not show significant change can support this explanation. If this hypothesis is true, although the blood pressure elevation has been achieved by phenylephrine administration during reduced preload, it means that it is actually supported by the afterload increase and the consequential increase in the contractility (owing to the reflective coronary flow increase). Then it could be possible for us to assess that this might be an inappropriate state that lacks the preload replacement. And we could also state that Ees, unlike CI, shows pure myocardial contractility. This is a desirable hypothesis that we would like to choose. Nevertheless, we think there might be a complex compensatory effect caused by bleeding and the afterload-dependent increase in coronary flow.

Thirdly, the calculation method may unfortunately have contributed to the result. Since Ea was calculated as Pes divided by SV, it naturally increased with rising arterial pressure. The Ees was obtained by multiplying Ea by the Ees/Ea ratio, which tends to exceed 1 under hemorrhagic shock conditions. In this situation, both Ea and Ees fluctuated in the same direction, potentially leading to an apparent increase in Ees that did not fully reflect the actual hemodynamic state.

Unfortunately, we must also address whether our method is suitable for assessing heart contractility. Boly et al. observed Ees derived from mean arterial pressure measured via arterial pressure line insertion and left ventricular end-systolic volume measured by TEE [16]. They presented a theory for estimating Ees from these measurements using the IVC compression method. Their study aimed to compare the IVC compression method with an estimation technique using phenylephrine injection. They concluded that while phenylephrine injection can estimate Ees, it cannot replace values obtained from the IVC compression method. They also pointed out that Ees assessment remains challenging because the highly invasive IVC compression method cannot be applied for Ees measurement in routine surgery. Unlike our approach, their study did not use approximations. In contrast, our method required the approximation of V_0_ = 0, making the evaluation of the heart contractility even more difficult than their approach. We think we have to conduct a study comparing the Ees obtained from MAP and TEE, which we are now working on.

### 4.4. Implications and Future Directions

In this study, invasive procedures such as the insertion of an esophageal stethoscope and an arterial line were required. We are considering ways to reduce the invasiveness of these procedures for future research; that is, the esophageal stethoscope could be replaced by a transthoracic phonography device, the arterial line by a cuff-based blood pressure measurement, and the SV by estimating the esSV using a transcutaneous oxygen saturation (SpO_2_) device. These modifications would enable a less invasive measurement approach.

The esSV can currently be measured using a noninvasive continuous cardiac output monitor (esCCO, Nihon Kohden). This device calculates cardiac output using pulse wave transit time. To date, esCCO has been shown to correlate highly with CO measured using a thermodilution catheter [17]. We believe that the SV measured by esCCO should also correlate with the SV measured by a thermodilution catheter, and we are concurrently evaluating the reliability of clinical data, such as esSV and esSVV, calculated by esCCO. Transthoracic phonography devices are still being reviewed by the ethics committee of our institution. Although the issue of potential leakage current and its impact on the human body is unlikely to pose a problem, some concerns remain under consideration; therefore, they have not yet been introduced. These trials to make all methods less invasive leads to an easy assessment of the left ventricular pressure–volume loop in daily clinical cases. As mentioned earlier, unnecessary insertion of TEE or Swan–Ganz catheters into patients is not recommended due to the invasive nature of the procedure. And these days, alternative devices such as Flo Trac sensors or other hemodynamic measurement devices can easily be applied to patients due to their noninvasiveness. If we do not need to insert any invasive devices into patients to understand their hemodynamics, the method can easily to apply to every kind of patient, even if they are not in the operating room, such as ICU patients or emergency patients. Even patients outside the hospital can gain the benefits of analyzing hemodynamics. In summary, one major objective of this research is to develop less invasive measurement methods and expand the scope of application for assessing patient hemodynamics. Another significant goal is that, as case numbers accumulate, our method will contribute to decisions regarding fluid and blood transfusion versus vasopressor administration in hemorrhagic shock patients, and to the choice of vasopressor—whether phenylephrine or norepinephrine is preferable. Furthermore, we anticipate that decision-making using our method will lead to improved clinical outcomes, such as reduced risks of postoperative complications like renal failure and cardiovascular events.

### 4.5. Limitations

This study has several limitations. The Ees/Ea was calculated under the assumption of preserved cardiac function. However, left ventricular unstressed volume (V_0_) is known to increase in patients with impaired cardiac function. Since we assumed V_0_ = 0 in the calculation, this method may not be appropriate for such patients. In cases where V < B-> cannot be assumed to be zero, Ees can be estimated using central venous pressure (CVP). Ea was calculated from Pes and SV, and EDV was considered to correlate with the left ventricular stressed volume (Vs). VS can be estimated by a lumped parameter model using CVP, and EDV can then be derived from Vs. Ees can be obtained from the equation EDV = Pes/Ees + SV. Thus, if CVP can be measured, it may be possible to evaluate Ees, Ea, and EDV, even in patients with impaired cardiac function. However, this issue requires further investigation. From the perspective of invasiveness, a noninvasive venous pressure-monitoring device (ezCVP, Nihon Kohden) that estimates CVP from cuff-based blood pressure measurements has been developed. Its use could enable the measurement of Ees, Ea, and EDV while maintaining low invasiveness.

Patients with atrial fibrillation, atrial premature contractions, or ventricular premature contractions present another challenge because the measurements of SV, PEP, and ET may vary and become inaccurate, thereby reducing reliability. Consequently, obtaining accurate measurements is difficult in such cases. However, changing the evaluation method would allow reliable data to be obtained.

In the FloTrac system, SV is updated every 20 s and displayed as the average value over the preceding minute [18]. Although the beat-to-beat values lack accuracy, the 1 min average demonstrates reasonable reliability and may be useful for trend evaluation, such as for CI. Therefore, even in patients with arrhythmias, the use of averaged data over 1 min or longer intervals may allow the assessment of Ees/Ea with acceptable accuracy.

Similarly, PEP and ET measurements may also be inaccurate in patients with arrhythmias (ET may occasionally appear as a negative value). However, if these parameters are assessed not on a beat-to-beat basis but as moving averages over several minutes, they may still provide reasonably reliable indicators.

Although this method has several limitations, many of them can be addressed in future studies. The calculations for Ees, Ea, and EDV could therefore offer substantial clinical value. The results of this study and how they can be interpreted from the perspective of previous studies and working hypotheses should be explored. The findings and their implications should be discussed in the broadest possible context.

### 4.6. Perspective

Finally, it should be noted that this research does not advocate replacing fluid therapy or blood transfusion therapy with vasopressors such as phenylephrine. Cao stated that, “Balanced transfusion characterized by the transfusion of red blood cells, fresh frozen plasma, and platelets equivalent to the constitution of whole blood is a cornerstone in the management of active bleeding and hemorrhagic shock in obstetric and nonobstetric patients.” [19]. No such paper contains statements supporting intervention with phenylephrine alone. Furthermore, while Meng states that phenylephrine remains a solid option for managing hypotension and maintaining target blood pressure, it also suggests that in certain patients, such as those with low-cardiac-output heart failure, dosage should be carefully considered or alternative therapies explored [20]. We have to broadly consider physiology, and to optimize patients’ hemodynamics, we should use both infusion therapy or transfusion therapy and vasopressors effectively in order to maintain organ perfusion.

## 5. Conclusions

Phenylephrine administration to patients with reduced intravascular volume due to hemorrhage demonstrated increased afterload, as calculated from Ea, and increased cardiac contractility, as calculated from Ees. Our noninvasive method for calculating EDV, Ea, and Ees has the potential to be valuable for monitoring the hemodynamics of patients under anesthesia.

## Figures and Tables

**Table 1:** Patients’ characteristics.

Characteristics.	Value
Age, years	61 ± 9
Gender, male/female, %	43/57
Height, cm	160 ± 6
Weight, kg	60 ± 3
BMI, kg/m^2^	23 ± 3
HR, bpm	76 ± 12
MAP, mmHg	67 ± 11
SVV, %	11 ± 3
SVRI, dynes·s/cm^5^/m^2^	1540 ± 310
CI, L/min/m^2^	2.6 ± 0.4
EDV, mL/m^2^	49 ± 6.3
Ea, mmHg·m^2^/mL	2.2 ± 0.4
Ees, mmHg·m^2^/mL	5.5 ± 1.4
SV, mL	54 ± 7
PEP, second	87 ± 12
ET, second	298 ± 16

BMI, body mass index; CI, cardiac index; Ea, effective arterial elastance; EDV, end-diastolic volume; Ees, end-systolic elastance; ET, ejection time; HR, heart rate; MAP, mean arterial pressure; PEP, pre-ejection period; SV, stroke volume; SVRI, systemic vascular resistance index.

**Table 2:** Results for linear mixed-effect model analysis. For all models, age, gender, blood loss volume, and presence of hypertension were adjusted as covariates, and patient ID was included as a random effect.

Dependent Variables	β (Time)	SE	t Value	*p* Value	Marginal R^2^	Conditional R^2^
HR, bpm	−2.0	1.9	−1.1	0.30	0.09	0.88
MAP, mmHg	8.7	2.7	3.3	<0.01	0.13	0.60
SVV, %	−2.0	0.76	−2.7	0.01	0.14	0.71
SVRI, dynes·s/cm^5^/m^2^	200	57	3.5	<0.01	0.35	0.78
CI, L/min/m^2^	−0.03	0.09	−0.39	0.7	0.08	0.73
EDV, mL/m^2^	0.18	1.7	0.11	0.91	0.30	0.40
Ea, mmHg·m^2^/mL	0.22	0.11	2.1	<0.05	0.12	0.31
Ees, mmHg·m^2^/mL	0.73	0.36	2.0	<0.05	0.29	0.45

## Data Availability

The data that support the findings of this study are available from the corresponding author upon reasonable request.

## References

[1] Wesselink E.M., Kappen T.H., Torn H.M., Slooter A.J.C., van Klei W.A. (2018). Intraoperative hypotension and the risk of postoperative adverse outcomes: A systematic review. Br. J. Anaesth..

[2] Futier E., Lefrant J.Y., Guinot P.G., Godet T., Lorne E., Cuvillon P., Bertran S., Leone M., Pastene B., Piriou V. (2017). Effect of individualized vs standard blood pressure management strategies on postoperative organ dysfunction among high-risk patients undergoing major surgery: A randomized clinical trial. JAMA.

[3] Scheuren K., Wente M.N., Hainer C., Scheffler M., Lichtenstern C., Martin E., Schmidt J., Bopp C., Weigand M.A. (2009). Left ventricular end-diastolic area is a measure of cardiac preload in patients with early septic shock. Eur. J. Anaesthesiol..

[4] Vincent J.L., Rhodes A., Perel A., Martin G.S., Rocca G.D., Vallet B., Pinsky M.R., Hofer C.K., Teboul J.L., Boode W.P. (2011). Update on hemodynamic monitoring: A consensus of 16. Crit. Care.

[5] Hayashi K., Shigemi K., Shishido T., Sugimachi M., Sunagawa K. (2000). Single-beat estimation of ventricular end-systolic elastance-effective arterial elastance as an index of ventricular mechanoenergetic performance. Anesthesiology.

[6] Russell D.W., Casey J.D., Gibbs K.W., Ghamande S., Dargin J.M., Vonderhaar D.J., Joffe A.M., Khan A., Prekker M.E., Brewer J.M. (2022). Effect of fluid bolus administration on cardiovascular collapse Among critically ill patients undergoing tracheal intubation: A randomized clinical trial. JAMA.

[7] Kappus R.M., Ranadive S.M., Yan H., Lane A.D., Cook M.D., Hall G., Harvey I.S., Wilund K.R., Woods J.A., Fernhall B. (2013). Validity of predicting left ventricular end systolic pressure changes following an acute bout of exercise. J. Sci. Med. Sport.

[8] Lonjaret L., Lairez O., Minville V., Geeraerts T. (2014). Optimal perioperative management of arterial blood pressure. Integr. Blood Press. Control.

[9] Du Bois D., Du Bois E.F. (1916). A formula to estimate the approximate surface area if height and weight be known. Arch. Intern. Med..

[10] Richards E., Lopez M.J., Maani C.V. (2023). Phenylephrine. StatPearls.

[11] Hooper N., Armstrong T.J. (2022). Hemorrhagic shock. StatPearls.

[12] Cannesson M., Jian Z., Chen G., Vu T.Q., Hatib F. (2012). Effects of phenylephrine on cardiac output and venous return depend on the position of the heart on the Frank-Starling relationship. J. Appl. Physiol. (1985).

[13] Højlund J., Cihoric M., Foss N.B. (2024). Vasoconstriction with phenylephrine increases cardiac output in preload dependent patients. J. Clin. Monit. Comput..

[14] Giraud R., Siegenthaler N., Arroyo D., Bendjelid K. (2014). Impact of epinephrine and norepinephrine on two dynamic indices in a porcine hemorrhagic shock model. J. Trauma Acute Care Surg..

[15] Sequeira V., Maack C., Reil G.-H., Reil J.-C. (2025). The Anrep effect in septic shock: A mechanism of cardiac adaption. Br. J. Anesth..

[16] Boly I., Picod A., Guerin A., Argaud T., Senat T., Fellahi J.L., Jacquet-Lagrèze M. (2013). Minimally invasive intraoperative estimation of left-ventricular end-systolic elastance with phenylephrine as loading intervention. Br. J. Anaesth..

[17] Yamada T., Tsutsui M., Sugo Y., Sato T., Akazawa T., Sato N., Yamashita K., Ishihara H., Takeda J. (2012). Multicenter study verifying a method of noninvasive continuous cardiac output measurement using pulse wave transit time: A comparison with intermittent bolus thermodilution cardiac output. Anesth. Analg..

[18] Ji F., Li J., Fleming N., Rose D., Liu H. (2014). Reliability of a new 4th generation FloTrac algorithm to track cardiac output changes in patients receiving phenylephrine. J. Clin. Monit. Comput..

[19] Cao D., Arens A.M., Chow S.L., Easter S.R., Hoffman R.S., Lagina A.T., Lavonas E.J., Patil K.D., Sutherland L.D., Tijssen J.A. (2025). Part 10: Adult and Pediatric Special Circumstances of Resuscitation: 2025 American Heart Association Guidelines for Cardiopulmonary Resuscitation and Emergency Cardiovascular Care. Circulation.

[20] Meng L., Sun Y., Zhao X., Meng D.M., Adams D.C., McDonagh D.L., Rasmussen M. (2024). Effects of phenylephrine on systemic and cerebral circulations in humans: A systematic review with mechanistic explanations. Anesthesia.

